# Earliest activation time is a good predictor of successful ablation of idiopathic outflow tract ventricular arrhythmias

**DOI:** 10.1002/clc.23578

**Published:** 2021-02-20

**Authors:** Ji‐Hoon Choi, Hee‐Jin Kwon, Hye Ree Kim, Seung‐Jung Park, June Soo Kim, Young Keun On, Kyoung‐Min Park

**Affiliations:** ^1^ Division of Cardiology, Department of Medicine, Samsung Medical Center Sungkyunkwan University School of Medicine Seoul Republic of Korea

**Keywords:** catheter ablation, local activation time, mapping, premature ventricular complex, ventricular arrhythmia

## Abstract

**Background:**

In idiopathic outflow tract ventricular arrhythmias (OT‐VAs), identifying the site with the earliest activation time (EAT) using activation mapping is critical to eliminating the arrhythmogenic focus. However, the optimal EAT for predicting successful radiofrequency catheter ablation (RFCA) has not been established.

**Hypothesis:**

To evaluate the association between EAT and successful RFCA in idiopathic OT‐VAs and to determine the optimal cut‐off value of EAT for successful ablation.

**Methods:**

We retrospectively analyzed patients undergoing RFCA for idiopathic OT‐VAs at a single center from January 2015 to December 2019.

**Results:**

Acute procedural success was achieved in 168 patients (87.0%). Among these patients, 158 patients (81.9%) were classified in the clinical success group according to the recurrence of clinical VAs during median (Q1, Q3) follow‐up (330 days [182, 808]). EAT was significantly earlier in the clinical success group compared with the recurrence (*p* = .006) and initial failure (*p* < .0001) groups. The optimal EAT cut‐off value predicting clinical success was −30 ms in the right ventricular outflow tract (RVOT) with 77.4% sensitivity and 96.4% specificity. In all cases of successful ablation in the left ventricular outflow tract (LVOT), EAT in the RVOT was not earlier than −29 ms.

**Conclusions:**

EAT in patients with successful catheter ablation was significantly earlier than that in patients with recurrence and initial failure. EAT earlier than −30 ms could be used as a key predictor of successful catheter ablation as well as an indicator of the need to shift focus from the RVOT to the LVOT.

## 1 INTRODUCTION

Idiopathic outflow tract ventricular arrhythmias (OT‐VAs) are the most common anatomical subtype of idiopathic VA and typically occur in healthy patients without clinically apparent structural heart disease. The main mechanism of idiopathic OT‐VAs is cyclic AMP‐mediated triggered activity and arrhythmogenic focus is usually presented as a focal source.^1^ Thus, finding the site of origin largely depends on activation mapping rather than pace mapping. In addition, identifying the site with the earliest activation time (EAT) is pivotal for eliminating the arrhythmogenic substrate. Generally, the site with presystolic activation over 20 ms and QS unipolar morphology is considered the possible site of origin of VAs.^2^, ^3^ However, the optimal EAT to predict successful radiofrequency catheter ablation (RFCA) has not been determined. Therefore, the purpose of this study was to investigate the association between successful RFCA and EAT and to identify the precise cut‐off value predicting successful ablation in patients undergoing catheter ablation for OT‐VAs.

## 2 METHODS

### 2.1 Study population

From January 2015 to December 2019, a total of 219 consecutive patients who underwent RFCA for OT‐VAs at Samsung Medical Center were evaluated for eligibility. Echocardiography was reviewed to exclude patients with congenital heart disease, ischemic cardiomyopathy, non‐ischemic dilated cardiomyopathy, hypertrophic cardiomyopathy, arrhythmogenic right ventricle (RV) dysplasia, valvular heart disease, and previous cardiac surgery. Indications for RFCA in patients with idiopathic OT‐VAs were as follows: (1) Symptomatic frequent premature ventricular complexes (PVCs) (≥15% of all beats on Holter monitoring) despite adequate antiarrhythmic agents over 2 months, (2) Symptomatic drug‐resistant non‐sustained ventricular tachycardia (VT), (3) Sustained VT, and (4) PVC‐induced cardiomyopathy. Informed consent was obtained from all patients before study enrollment. This study complied with the Declaration of Helsinki, and the research protocol was approved by the local institutional ethics board.

### 2.2 Electrophysiologic study and RFCA

All electrophysiologic study (EPS) were conducted in a fasting state without general anesthesia or sedation in patients who discontinued antiarrhythmic drugs for at least five half‐lives before the study. A surface 12‐lead electrocardiogram (ECG) and intracardiac electrogram (EGM) were simultaneously displayed and recorded using the Cardiolab™ electrophysiology recording system (GE Healthcare, Chicago, IL). After local anesthesia, a 6‐Fr His‐RV electrode catheter was advanced through a right femoral vein to the RV apex and His bundle region. Mapping and RFCA were performed using a 8‐Fr quadripolar deflectable catheter with a 3.5‐mm open‐irrigated‐tip (Thermocool SF NAV mainly in the early period, and Thermocool SmartTouch‐SF catheter after 2018, Biosense Webster, Diamond Bar, CA) introduced through a long sheath via a right femoral vein. In the case of left ventricular outflow tract (LVOT), we used a retrograde aortic approach via the right femoral artery.

To identify the earliest site of endocardial activation, a 3‐dimensional electroanatomic mapping system (Carto3, Biosense Webster) was used for activation mapping. Pace mapping was also performed at the site of earliest activation and the matching score was presented as a percentage using an automated ECG matching program (PASO, Biosense Webster). EAT was measured from the initial deflection on distal bipolar EGM to the earliest onset of PVC‐QRS on the 12‐lead surface ECG (Figure S1). Except for cases with right bundle branch block (RBBB) morphology of clinical VAs, we usually began by mapping the right ventricular outflow tract (RVOT). If a site with EAT earlier than −20–−25 ms could not be identified in the RVOT, the LVOT was mapped.

Radiofrequency (RF) applications were delivered initially at the earliest site of endocardial activation with a maximal power of 25–40 W using an RF generator (Stockert, Biosense Webster). RF output was upward titrated independently from 25 to 40 W according to impedance and a temperature limit under 50°C. If clinical VAs were suppressed within 20–30 seconds, RF application was maintained for 60–90 seconds. Booster burn was always applied at the successful ablation site for 60 seconds to completely eliminate the arrhythmogenic substrate.

### 2.3 12‐lead ECG analysis of the origin of OT‐VA

Five 12‐lead ECG algorithms including R‐wave duration index, R/S‐wave amplitude index, R‐wave deflection interval combined with R‐wave amplitude index, V_2_S/V_3_R index, and V_2_ transition ratio were used to predict the origin of OT‐VA. The following ECG parameters were measured during clinical VA (PVC/VT) using a digital caliper on the Cardiolab™ recording system: (1) R‐wave duration in leads V_1_–V_2_; (2) R‐ and S‐wave amplitude in leads V_1_–V_3_; (3) R‐ and S‐wave amplitude in lead V_2_ during sinus rhythm; (4) R‐wave deflection interval in lead V_3_, and (4) Total QRS duration. We evaluated the predictive accuracy of these ECG algorithms for differentiating right from left OT‐VA origin in patients with V_3_ precordial transition of clinical OT‐VA.

### 2.4 Definitions

Clinical VA was defined as a PVC and/or VT with the morphology most frequently observed in preprocedural Holter monitoring and 12‐lead ECG. Acute procedural success was defined as no detection of any clinical VA during a waiting period (≥30 minutes) after catheter ablation under a standardized stimulation protocol. Clinical success was defined as the complete elimination of clinical VT during the follow‐up period and/or ≥80% reduction of clinical PVC on postprocedural Holter monitoring and regular 12‐lead ECG. Reappearance of clinical VT and/or a <80% reduction in clinical PVC after acute procedural success was classified as recurrence. “Initial failure” indicated a case that did not achieve acute procedural success, while “no clinical success” was used to describe both initial failure and recurrence.

### 2.5 Follow‐up

All patients were scheduled to visit the outpatient clinic 1 month after the procedure and at 3–4‐month intervals thereafter for 12‐lead ECG. Holter monitoring was performed 3–6 months after the procedure. Patients with recurrent symptoms were evaluated immediately using Holter or event monitoring regardless of follow‐up interval. In the case of follow‐up loss, we contacted patients over the telephone to determine whether symptoms or VAs had recurred and encouraged patients to resume follow‐up.

### 2.6 Statistical analysis

Continuous variables are expressed as mean with standard deviation (SD) or median with 25th (Q1) and 75th (Q3) percentiles according to normality test, and categorical variables as the number with a percentage. To compare two groups, unpaired Student's *t*‐test and Mann–Whitney test were performed for continuous variables and Pearson's *χ* ^2^ and Fisher's exact test for categorical variables. A comparison of continuous variables among the three groups was analyzed with Kruskal–Wallis test, and post hoc analysis was conducted with Dunn's multiple comparisons test. To evaluate sensitivity, specificity, the area under the curve (AUC), and the optimal cut‐off value for predicting clinical success, receiver operating characteristic (ROC) analysis was used with DeLong's test to compare two ROC curves. Statistical significance was defined as a two‐tailed *p* < .05. All statistical analyses were performed using SPSS statistical software version 25.0 (IBM, Armonk, NY).

## 3 RESULTS

### 3.1 Baseline characteristics

Over the 5‐year study period, a total of 219 patients underwent catheter ablation for OT‐VAs. Twenty‐six patients were excluded according to the exclusion criteria, and a total of 193 patients (mean age 48 ± 13 years; female 63%) were included in the study group (Figure S2). Patients were divided into two groups based on clinical success. The clinical characteristics of the study cohort are summarized in Table 1. The clinical VAs demonstrated PVC in 126 patients (65.3%), non‐sustained VT in 48 (24.9%), and sustained VT in 19 (9.8%). The median (Q1, Q3) PVC burden was 26.0% (15.1, 34.0) and 23 patients (11.9%) had PVC‐induced CMP with a median (Q1, Q3) LV ejection fraction of 45.0% (36.7, 47.1). Only 12 patients (6.2%) exhibited PVC/VT with an RBBB morphology (R/S wave ratio in lead V_1_ > 1) on preprocedural 12‐lead ECG. The precordial transition zone was V_4_–V_6_ in 129 patients (66.8%), V_3_ in 46 (23.8%), and V_1_–V_2_ in 18 (9.3%). There were no significant differences in age, sex, underlying disease, left ventricular (LV) systolic function, the proportion of clinical arrhythmia, or ablation‐related variables between the two groups except for EAT.

**TABLE 1:** Baseline characteristics of the study population

	All (*N* = 193)	Clinical success (*N* = 158)	No clinical success (*N* = 35)	*p* value
Age, years	48 ± 13	48 ± 13	52 ± 15	.103
Female sex, *n* (%)	121 (62.7)	102 (64.6)	19 (54.3)	.256
BMI, kg/m^2^	23.8 (21.6, 25.8)	23.8 (21.6, 25.7)	23.4 (21.6, 27.5)	.925
Hypertension, *n* (%)	50 (25.9)	43 (27.2)	7 (20.0)	.378
Diabetes, *n* (%)	15 (7.8)	14 (8.9)	1 (2.9)	.314
Coronary artery disease, *n* (%)	4 (2.1)	3 (1.9)	1 (2.9)	.554
Hemoglobin, g/dl	13.4 ± 1.4	13.4 ± 1.4	13.5 ± 1.4	.746
Creatinine, mg/dl	0.72 (0.63, 0.85)	0.71 (0.62, 0.86)	0.74 (0.66, 0.82)	.777
LVEF, %	59.0 (56.0, 63.0)	59.0 (56.0, 63.0)	59.0 (55.0, 65.0)	.943
LVEDD, mm	51.2 ± 5.8	51.1 ± 5.9	52.0 ± 5.3	.414
LVESD, mm	32.0 (29.0, 36.4)	32.0 (28.6, 36.5)	32.0 (29.0, 36.0)	.853
Clinical arrhythmias, *n* (%)				.842
PVC only	126 (65.3)	104 (65.8)	22 (62.9)	
Non‐sustained VT	48 (24.9)	38 (24.1)	10 (28.6)	
Sustained VT	19 (9.8)	16 (10.1)	3 (8.6)	
PVC burden, %	26.0 (15.1, 34.0)	27.0 (15.6, 34.5)	20.0 (14.0, 33.0)	.321
PVC‐induced CMP, *n* (%)	23 (11.9)	17 (10.8)	6 (17.1)	.384
LBBB morphology, *n* (%)	181 (93.8)	149 (94.3)	32 (91.4)	.459
RBBB morphology, *n* (%)	12 (6.2)	9 (5.7)	3 (8.6)	
Precordial transition, *n* (%)				.557
V_1_–V_2_	18 (9.3)	14 (8.9)	4 (11.4)	
V_3_,	46 (23.8)	40 (25.3)	6 (17.1)	
V_4_–V_6_	129 (66.8)	104 (65.8)	25 (71.4)	
QRS duration, ms	144 (133, 158)	144 (133, 159)	146 (133, 153)	.490
Total RF application, *n*	5 (4, 9)	5 (4, 8)	7 (4, 9)	.203
Total RF application time, s	307 (189, 467)	297 (188, 454)	344 (232, 501)	.368
Earliest activation time, ms	−30 (−25, −31)	−30 (−28, −32)	−22 (−18, −24)	<.0001

*Note*: Values are given as mean ± SD, n (%), or median (Q1, Q3).

Abbreviations: BMI, body mass index; CMP, cardiomyopathy; LBBB, left bundle branch block; LVEDD, left ventricular end diastolic dimension; LVEF, left ventricular ejection fraction; LVESD, left ventricular end systolic dimension; PVC, premature ventricular complex; RBBB, right bundle branch block, RF, radiofrequency; VT, ventricular tachycardia.

### 3.2 RFCA outcomes

Acute procedural success was achieved in 168 patients (87.0%), while 25 patients (13.0%) showed failed RFCA. Among the acute procedural success group, 10 patients (6.0%) experienced a recurrence of clinical VAs during median (Q1, Q3) follow‐up (330 days [182, 808]). The RVOT accounts for nearly 80% of site of origin. The septal wall of the RVOT (77.4%) was the most common arrhythmogenic focus in the RVOT and the aortic cusp (85.3%) was the most common in the LVOT (Table S1). Nine patients with RBBB morphology PVC/VT had a successful ablation in the LVOT. One in five patients (20%) with PVC/VT transition in lead V_2_ had a successful ablation at the RVOT, and five in 104 patients (4.8%) with PVC/VT transition ≥lead V_4_ had a successful ablation at the LVOT (Figure S3). There was no major complication after RFCA, except for one case with arteriovenous fistula at vascular access.

### 3.3 Predictive value of EAT

**FIGURE 1:**
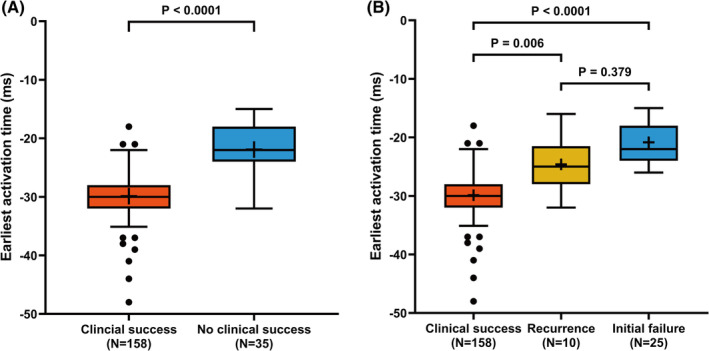
Box plot of the earliest activation time in each study group. The height of the box indicates the interquartile range (IQR), the horizontal bar within the box indicates the median, the cross within the box indicates the mean, the whiskers indicate 1.5 times the IQR, and the circles indicates outliers. (A) Clinical success versus no clinical success. (B) Clinical success versus recurrence versus initial failure

Comparisons of EAT between two groups (clinical success vs. no clinical success) and among three groups (clinical success vs. recurrence vs. initial failure) are shown in Figure 1. The median (Q1, Q3) EAT of the clinical success group was significantly earlier compared with the no clinical success group (−30 ms [−28, −32] vs. −22 ms [−18, −24]; *p* < .0001). There was a significant difference in median (Q1, Q3) EAT among the three groups (*p* < 0.0001); moreover, post‐hoc analysis showed that EAT was significantly earlier in the clinical success group than in the recurrence group (−30 ms [−28, −32] vs. −25 ms [−22, −28]; *p* = .0006) or the initial failure group (−30 ms [−28, −32] vs. −22 ms [−18, −24]; *p* < .0001). However, there was no significant difference in median EAT between the recurrence group and initial failure group (*p* = .379).

**FIGURE 2:**
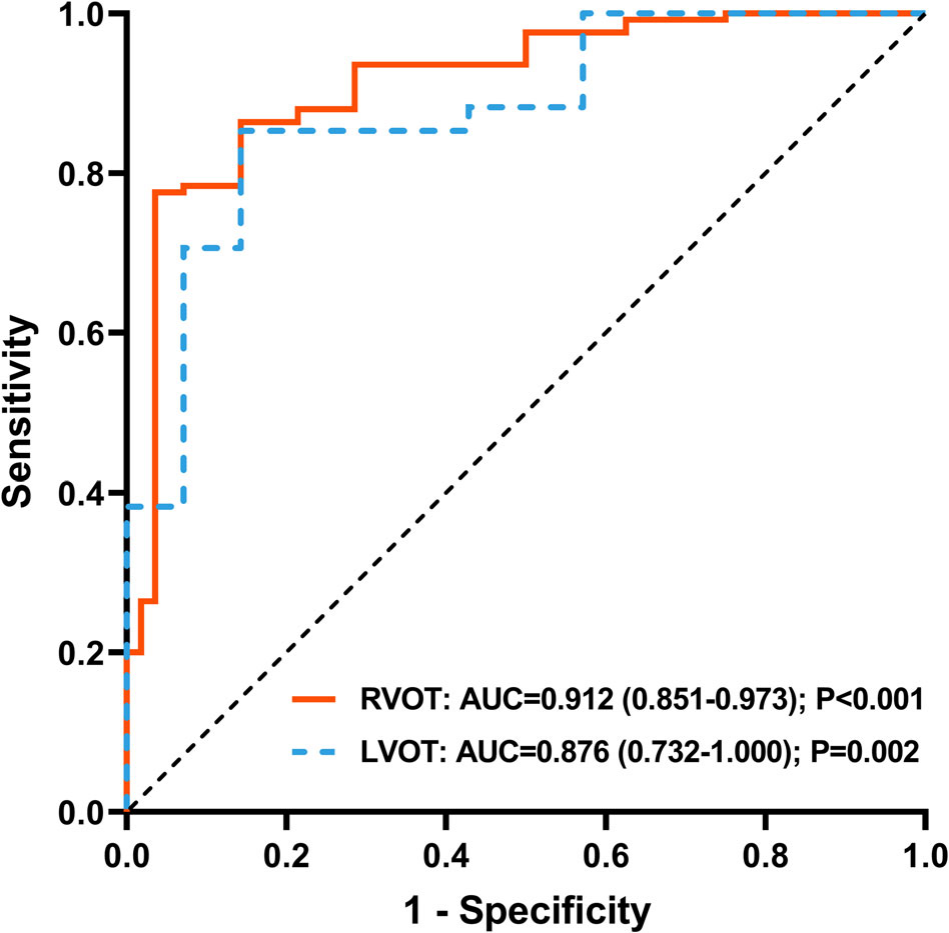
Receiver operating characteristic curves for the earliest activation time predicting clinical success. Abbreviations: AUC, area under the curve; RVOT, right ventricular outflow tract; LVOT, left ventricular outflow tract

The area under the ROC curve for EAT predicting clinical success in the RVOT was 0.912 (95% CI 0.851–0.973; *p* < .001), while that in the LVOT was 0.876 (95% CI 0.732–1.000; *p* = .002) (Figure 2). There was no significant difference in AUC between the two ROC curves (*p* = .636). The optimal cut‐off value of EAT for predicting clinical success in the RVOT was −30 ms, which was characterized by 77.4% sensitivity and 96.4% specificity. Likewise, the optimal cut‐off value of EAT in the LVOT was −26 ms, which was characterized by 85.3% sensitivity and 85.7% specificity (Table 2).

**TABLE 2:** Diagnostic performance of EAT to predict clinical success of catheter ablation in the RVOT and LVOT

	EAT (ms)	Sensitivity (%)	Specificity (%)	PPV (%)	NPV (%)	ACC (%)
RVOT	−18	100.0	21.4	84.9	100.0	85.5
	−30	77.4	96.4	99.0	49.1	80.9
	−33	20.2	100.0	100.0	22.1	34.9
LVOT	−22	100.0	42.9	89.5	100.0	90.2
	−26	85.3	85.7	96.7	54.6	85.4
	−30	38.2	100.0	100.0	25.0	48.8

Abbreviations: ACC, accuracy; EAT, earliest activation time; LVOT, left ventricular outflow tract; NPV, negative predictive value; RVOT, right ventricular outflow tract; PPV, positive predictive value.

Figure S4 shows EAT in the RVOT in the clinical success group with an LBBB morphology of PVC/VT according to final successful ablation site. In all cases of successful ablation in the LVOT, EAT in the RVOT was not earlier than −29 ms. The best cut‐off value for EAT in the RVOT to differentiate an RVOT versus LVOT origin was −29 ms, with a sensitivity of 70.2% and a specificity of 100%.

## 4 DISCUSSION

### 4.1 Main findings

The aim of this study was to evaluate the association between EAT and successful RFCA in idiopathic OT‐VAs and to find the optimal cut‐off value of EAT for predicting successful RFCA. First, we found that EAT was significantly earlier in the clinical success group compared with the recurrence and initial failure groups. Second, the optimal cut‐off value for EAT could predict successful RFCA in the RVOT and LVOT with high accuracy. The results of this study have important clinical implications, as these findings will provide useful information to establish the optimal RFCA strategy during the procedure.

### 4.2 EAT as a predictor for successful ablation

In the past two decades, an improved understanding of the mechanism and anatomy of OT‐VAs and advances in electroanatomic mapping systems have made RFCA a standard therapy in drug‐refractory patients and those with a high PVC burden associated with reduced LV systolic function.^4^, ^5^ Given the complexity of outflow tract anatomy and its variations, precise electroanatomical mapping is crucial for successful RFCA. Pace mapping could be useful when spontaneous ventricular ectopies are not frequently induced enough to create detailed electroanatomic mapping.^6^ However, Yamada et al. reported that for VAs originating from the aortic root, pace mapping may not be accurate due to preferential conduction from the aortic root to the RVOT.^7^ In addition, Bogun et al. showed that the spatial resolution of pace mapping is inferior to that of activation mapping, and, more interestingly, pace mapping was inaccurate in nearly 20% of patients (three of 16 cases).^8^ Therefore, activation mapping is the preferred modality for identifying the site with EAT, and is usually consistent with the site of origin of VAs.

Some review articles concerning RFCA in OT‐VAs have suggested that a sharp bipolar signal preceding QRS onset by −20 to −30 ms usually indicates a successful ablation site.^2^, ^3^ However, there was no specific evidence supporting that opinion; in addition, conflicting study results about EAT have been reported. In a prospective study including 22 patients with idiopathic OT‐VAs, Guodong et al. reported that the local activation time of successful sites was significantly earlier compared with that of unsuccessful sites (−40 ± 10 ms vs. −28 ± 11 ms; *p* < .01) and suggested that a local activation time with −30 ms was the best cut‐off value for predicting successful ablation.^9^ In 2012, Yamada et al. reported that there was no difference in EAT between an initial success group and a failure group, but EAT predicted recurrence after successful ablation in a retrospective study with idiopathic RVOT‐VAs.^10^ These conflicting findings may be due to differences in the patient population, electroanatomic mapping modality, mapping catheter and technique, and detailed measurement methods. While our results showed a difference in EAT between the clinical success and no clinical success groups, a further large‐scale prospective study is needed to confirm the association between EAT and successful ablation.

### 4.3 EAT as a key element of ablation strategy

In patients without structural heart disease, the electrical activity caused by PVC is conducted through the normal myocardium; thus, it exhibits a characteristic ECG shape depending on the location of its occurrence.^11^ Several studies revealed relatively high accuracy for differentiating right from left OT‐VA origin on 12‐lead ECG algorithm.^12^, ^13^, ^14^, ^15^ However, the accuracy of prediction varies according to the patient population because there is considerable variability in lead positions, shape of the chest wall, heart rotation, and conduction properties from person to person.^16^, ^17^ In general, since the LVOT is posteriorly positioned compared with the RVOT, PVC originating from the LVOT tends to show a larger and wider R‐wave at lead V_1_ and V_2_. Therefore, when precordial transition is ≤lead V_2_, PVC is likely to originate from the LVOT; in contrast, when precordial transition is ≥lead V_4_, PVC is likely to originate from the RVOT.^15^ However, in cases with precordial transition in lead V_3_, it is difficult to discriminate between the RVOT and LVOT, and the accuracy of various ECG algorithms is relatively low.^11^ In our study, among patients who underwent successful ablation, 20% of those who had a successful ablation in the RVOT showed an early transition of PVC/VT in lead V_2_, while 4.8% of patients who had a successful ablation in the LVOT showed a late transition of PVC/VT (≥lead V_4_) (Figure S3). Moreover, in cases with PVC/VT transition in V_3_, various ECG algorithms showed low predictive performance for differentiating between the RVOT and LVOT (Table 3). Therefore, it is essential to establish an optimal ablation strategy because electroanatomic mapping is the only way to identify the precise site of origin. We always mapped the RVOT first except for cases with an RBBB PVC/VT morphology. In all cases of successful ablation in the LVOT, EAT in the RVOT was not earlier than −29 ms. Compared to various ECG algorithms, EAT in RVOT showed better predictive accuracy, with 75% sensitivity and 100% specificity. Therefore, we suggest EAT earlier than −30 ms as an element of ablation strategy that indicates the need to shift focus from the RVOT to the LVOT, as well as a good predictor of successful ablation. Based on the results of this study, in cases with an LBBB morphology of OT‐VA, we suggest that if EAT in the RVOT is not earlier than −30 ms, mapping of the LVOT should be done instead of catheter ablation in the RVOT.

**TABLE 3:** Diagnostic performance of EAT and various ECG algorithms in the clinical success group with V_3_ precordial transition of PVC/VT

	Sen (%)	Spe (%)	PPV (%)	NPV (%)	ACC (%)
EAT −29 ms in the RVOT	75.0	100.0	100.0	72.7	85.0
R‐wave duration index	75.0	62.5	75.0	62.5	70.0
R/S‐wave amplitude index	62.5	75.0	78.9	57.1	67.5
R‐wave deflection interval combined with R‐wave amplitude index	87.5	12.5	60.0	40.0	57.5
V_2_S/V_3_R index	83.3	68.8	80.0	73.3	77.5
V_2_ transition ratio	45.8	81.3	78.6	50.0	60.0

Abbreviations: ACC, accuracy; EAT, earliest activation time; ECG, electrocardiogram; NPV, negative predictive value; PPV, positive predictive value; PVC, premature ventricular complex; RVOT, right ventricular outflow tract; Sen, sensitivity; Spe, specificity; VT, ventricular tachycardia.

### 4.4 Limitations

There were several limitations to the current study. First, we did not routinely perform coronary venous system mapping or epicardial mapping in failed cases. Therefore, it is possible that EAT was underestimated and that the ablation site may not have represented the true origin of OT‐VA in failed cases. However, from another point of view, it may give us an important message that more detailed dense mappings are necessary to increases the success rate. Second, clinical success was determined based on relief of symptoms and 12‐lead ECG in 28 patients who did not undergo Holter monitoring after catheter ablation, including those lost to follow‐up. Third, as this study is a retrospective study, it is impossible to exclude the influence of unexamined confounding factors that may affect the study results. Thus, although our results showed that EAT is a good predictor of successful ablation, further multicenter prospective studies are warranted to confirm this result. Fourth, in some patients, PVC was not sufficiently induced during the procedure and activation mapping was performed incompletely.

## 5 CONCLUSIONS

EAT in patients with successful catheter ablation was significantly earlier than that in patients with recurrence and initial failure. EAT preceding QRS onset by −30 ms could be used to predict successful catheter ablation and as an indicator of the need to shift focus from the RVOT to the LVOT. If EAT is not earlier than −30 ms in the RVOT, mapping of LVOT should be performed before catheter ablation in the RVOT to determine the true origin of the OT‐VA and improve ablation outcomes.

## CONFLICTS OF INTEREST

The author declares that there is no conflict of interest that could be perceived as prejudicing the impartiality of the research reported.

## Supporting information


**Table S1.** Sites of successful RFCA in the clinical success group.
**Figure S1.** Measurement of the earliest activation time (EAT) and 3‐dimensional activation mapping of idiopathic OT‐VA in representative cases. (A) A patient presented with frequent PVCs despite antiarrhythmic drug treatment. Initial echocardiography revealed mild left ventricular systolic dysfunction (LVEF = 44%). Catheter ablation was performed in the RVOT mid septum where EAT was −35 ms. Acute procedural success was achieved and PVC burden was markedly reduced (99%) on follow‐up Holter monitoring. No recurrence was observed for more than 2 years. (B) A patient presented with repetitive non‐sustained VT and ablation was performed in the RVOT mid septum where EAT was −17 ms. Because PVC was rarely induced during the procedure, pace mapping was used. Thus, additional ablations were done at sites where pace mapping and clinical PVC were well‐matched after ablation at a site with an EAT of −17 ms. Two clinical PVCs were observed during the waiting period after catheter ablation. Follow‐up Holter monitoring showed one run of non‐sustained VT with a partially suppressed PVC burden (50%). Abbreviations: OT‐VA, outflow tract ventricular arrhythmia; PVC, premature ventricular complexes; LVEF, left ventricular ejection fraction; RVOT, right ventricular outflow tract; VT, ventricular tachycardia.
**Figure S2.** Study flow chart with the number of patients. Abbreviation: RV, right ventricular RFCA, radiofrequency catheter ablation.
**Figure S3.** The number of cases according to precordial transition in clinical success group. Abbreviations: RVOT, right ventricular outflow tract; LVOT, left ventricular outflow tract.
**Figure S4.** Scatter plot showing the earliest activation time in the RVOT in LBBB morphology OT‐VA. The solid lines within the plot indicate the median with the interquartile range. Abbreviations: RVOT, right ventricular outflow tract; LBBB, left bundle branch block; OT‐VA, outflow tract ventricular arrhythmia; LVOT, left ventricular outflow tract.
