# Supplementary material for: Earliest activation time is a good predictor of successful ablation of idiopathic outflow tract ventricular arrhythmias
Source: Clin Cardiol. 2021 Feb 20;44(4):573–9. doi: 10.1002/clc.23578 (PMC8027574; doi:10.1002/clc.23578)
Supplement: Supplementary file 1 — Table S1. Sites of successful RFCA in the clinical success group. Figure S1. Measurement of the earliest activation time (EAT) and 3‐dimensional activation mapping of idiopathic OT‐VA in representative cases. (A) A patient presented with frequent PVCs despite antiarrhythmic drug treatment. Initial echocardiography revealed mild left ventricular systolic dysfunction (LVEF = 44%). Catheter ablation was performed in the RVOT mid septum where EAT was −35 ms. Acute procedural success was achieved and PVC burden was markedly reduced (99%) on follow‐up Holter monitoring. No recurrence was observed for more than 2 years. (B) A patient presented with repetitive non‐sustained VT and ablation was performed in the RVOT mid septum where EAT was −17 ms. Because PVC was rarely induced during the procedure, pace mapping was used. Thus, additional ablations were done at sites where pace mapping and clinical PVC were well‐matched after ablation at a site with an EAT of −17 ms. Two clinical PVCs were observed during the waiting period after catheter ablation. Follow‐up Holter monitoring showed one run of non‐sustained VT with a partially suppressed PVC burden (50%). Abbreviations: OT‐VA, outflow tract ventricular arrhythmia; PVC, premature ventricular complexes; LVEF, left ventricular ejection fraction; RVOT, right ventricular outflow tract; VT, ventricular tachycardia. Figure S2. Study flow chart with the number of patients. Abbreviation: RV, right ventricular RFCA, radiofrequency catheter ablation. Figure S3. The number of cases according to precordial transition in clinical success group. Abbreviations: RVOT, right ventricular outflow tract; LVOT, left ventricular outflow tract. Figure S4. Scatter plot showing the earliest activation time in the RVOT in LBBB morphology OT‐VA. The solid lines within the plot indicate the median with the interquartile range. Abbreviations: RVOT, right ventricular outflow tract; LBBB, left bundle branch block; OT‐VA, outflow tract ventricular arrhythm [file CLC-44-573-s001.docx]

**Supplementary Table S1. Sites of successful RFCA in the clinical success group**

|  | Clinical success (n=158) |
| --- | --- |
| RVOT, n (%) | 124 |
| Septum | 96 (77.4) |
| Free wall | 28 (22.6) |
| Anterior | 67 (54.0) |
| Mid | 30 (24.2) |
| Posterior | 27 (21.8) |
| LVOT, n (%) | 34 |
| Aortic cusp | 29 (85.3) |
| LCC | 9 (26.5) |
| RCC | 6 (17.6) |
| NCC | 1 (3.0) |
| LCC-RCC junction | 12 (35.3) |
| LCC-NCC junction | 1 (3.0) |
| AMC | 5 (14.7) |

Abbreviations: RFCA, radiofrequency catheter ablation; RVOT, right ventricular outflow tract; LVOT, left ventricular outflow tract; LCC, left coronary cusp; RCC, right coronary cusp; NCC, non-coronary cusp; AMC, aorto-mitral continuity.

**Supplementary Figure S1** Measurement of the earliest activation time (EAT) and 3-dimensional activation mapping of idiopathic OT-VA in representative cases. (**A**) A patient presented with frequent PVCs despite antiarrhythmic drug treatment. Initial echocardiography revealed mild left ventricular systolic dysfunction (LVEF = 44%). Catheter ablation was performed in the RVOT mid septum where EAT was -35 ms. Acute procedural success was achieved and PVC burden was markedly reduced (99%) on follow-up Holter monitoring. No recurrence was observed for more than two years. (**B**) A patient presented with repetitive non-sustained VT and ablation was performed in the RVOT mid septum where EAT was -17 ms. Because PVC was rarely induced during the procedure, pace mapping was used. Thus, additional ablations were done at sites where pace mapping and clinical PVC were well-matched after ablation at a site with an EAT of -17 ms. Two clinical PVCs were observed during the waiting period after catheter ablation. Follow-up Holter monitoring showed one run of non-sustained VT with a partially suppressed PVC burden (50%). Abbreviations: OT-VA, outflow tract ventricular arrhythmia; PVC, premature ventricular complexes; LVEF, left ventricular ejection fraction; RVOT, right ventricular outflow tract; VT, ventricular tachycardia.

**Supplementary Figure S2** Study flow chart with the number of patients**.** Abbreviation: RV, right ventricular RFCA, radiofrequency catheter ablation.

**Supplementary Figure S3** The number of cases according to precordial transition in clinical success group. Abbreviations: RVOT, right ventricular outflow tract; LVOT, left ventricular outflow tract.

**Supplementary Figure S4** Scatter plot showing the earliest activation time in the RVOT in LBBB morphology OT-VA. The solid lines within the plot indicate the median with the interquartile range. Abbreviations: RVOT, right ventricular outflow tract; LBBB, left bundle branch block; OT-VA, outflow tract ventricular arrhythmia; LVOT, left ventricular outflow tract.
